# Immune-related key gene CLDN10 correlates with lymph node metastasis but predicts favorable prognosis in papillary thyroid carcinoma

**DOI:** 10.18632/aging.102780

**Published:** 2020-02-11

**Authors:** Zhaolan Xiang, Cheng Zhong, Aoshuang Chang, Junjun Ling, Houyu Zhao, Wei Zhou, Xianlu Zhuo

**Affiliations:** 1 Department of Otolaryngology, Southwest Hospital, Army Medical University, Chongqing, China; 2 Affiliated Hospital of Guiyang Medical University, Guiyang, China; 3 Chongqing Cancer Institute, Chongqing, China

**Keywords:** papillary thyroid carcinoma, CLDN10, immune cell infiltration, immunohistochemistry, bioinformatics method

## Abstract

The functions of immune cells in lymph node metastasis (LNM) have attracted considerable attention. This study aimed to screen the key immune-related and LNM-related genes in PTC. In the discovery phase, the immune-related genes in LNM were screened by using bioinformatics methods. In the validation phases, the association of the genes with LNM was first confirmed in a cohort from The Cancer Genome Atlas and a cohort based on a tissue chip. Then, the relationship of the genes with immune cell infiltration was further explored. Consequently, CLDN10 was identified, and its high expression was correlated with the presence of LNM in PTC but predicted a favorable prognosis. High CLDN10 expression was positively correlated with the infiltration of several immune cells, such as B cells, CD8+T cells, and macrophages. High CLDN10 expression may improve the outcomes of patients with PTC by increasing immune cell infiltration, although it might be associated with LNM. In conclusion, although CLDN1 might be correlated with LNM, it may also increase the infiltration of immune cells, including CD8+T cells and macrophages, and improve the clinical outcomes of patients with PTC. The effects of tumor purity and immune cell infiltration need to be considered in prognosis evaluation.

## INTRODUCTION

Thyroid cancer is the most common endocrine malignant disease in the world, with increasing incidence in recent decades. Thyroid cancer can be pathologically classified into well-differentiated and undifferentiated subtypes [1]. The former one mainly includes papillary thyroid cancer (PTC) and follicular thyroid cancer, whereas the latter one includes anaplastic thyroid cancer [2]. Of these subtypes, PTC is the most common one, constituting about 80% of all thyroid cancers [3].

Through standardized treatment, PTC often shows a favorable prognosis, with a 10-year survival rate accounting for 93% [4]. However, PTC is prone to metastasize to cervical lymph nodes, especially the central ones. Thus, a proportion of patients with PTC present unfavorable prognosis possibly because 20%–90% of PTC cases present with cervical lymph node metastasis (LNM) [5, 6]. Moreover, cervical LNM is the main risk factor associated with high recurrence in patients with PTC. In general, LNM occurs in the central region and then extends to the lateral region. In this process, skip metastasis can be observed [7]. Hence, the molecular mechanisms underlying LNM must be elucidated to develop preventive and treatment strategies.

To date, a number of studies have investigated the contributions of microenvironment and immune cell infiltration to cancer development. Cancer tissues contain not only cancer cells but also noncancer cells, such as stromal and immune cells. Noncancer cells dilute the purity of cancer cells and serve critical functions in cancer biology. Under different purity conditions, recognized predictive indicators are no longer effective [8]. Thus, the composition and proportion of stromal and immune cells in cancers may determine the clinical outcomes of patients. In colon cancer, low tumor purity is correlated with poor prognosis because of high mutation frequency in key pathways and purity-related microenvironmental changes [9]. In PTC, immune cell infiltrates are correlated with lymph node N stage [10]. In these biological processes, immune-related genes may influence the prognosis of cancer patients by affecting the abundance of infiltrating immune cells [11]. Thus, the immune-related genes in a certain phenotype of cancers must be identified to elucidate the exact mechanisms and find biomarkers or targets for cancer diagnosis and therapy.

In the present study, we aimed to find the key immune-related genes in LNM of PTC and further assess their functions in cancer progression. The study included three phases: a discovery phase and two validation phases. In the discovery phase, the immune-related genes that had a close association with LNM in PTC were screened by using bioinformatic methods. In the validation phases regarding LNM, the relationships of the screened genes with LNM and prognosis were evaluated in a The Cancer Genome Atlas (TCGA) cohort and in a cohort based on a tissue chip, respectively. In the validation phase regarding immunity, the association of CLDN10 expression with immune cell infiltration levels was assessed.

CLDN10 was identified as the key gene. Its high expression was correlated with LNM, but it might be a good indicator for PTC prognosis. The discrepancy overturned our long-held belief that the presence of LNM is associated with poor survival, implicating the critical contributions of immune cell infiltration to PTC development.

## RESULTS

### Relationship of immune and stromal scores with clinical features

The immune and stromal scores and the clinical information of the TCGA cohort were obtained. The characteristics of the PTC cohort are presented in Table 1. The immune and stromal scores were respectively divided into high and low groups on the basis of their median levels. As shown in Table 2, high immune scores showed a significant association with advanced clinical stages, high T stages, and LNM (P<0.05). Although the comparisons of stromal scores were not significant, a borderline association was shown in the LNM comparison (P=0.07). The data indicated that both immune and stromal scores might have a correlation with LNM in PTC.

**Table 1:** Patient characteristics from TCGA database.

**Characteristic**		**No. of patients**
Age (year)	509	
Median (range)		46 (15-89)
<45		233
≥45		276
Gender	509	
Male		139
Female		370
Historical type	509	
classical		365
Follicular		102
Tall cell		35
Other		7
Clinical stage	507	
I		288
II		51
III		111
IV		57
T stage	509	
T1		142
T2		168
T3		174
T4		23
Tx		2
N stage	509	
N0		226
N1		233
Nx		50
Distant metastasis	508	
M0		283
M1		9
Mx		216

**Table 2:** Relationship between Immune and Stromal scores and clinicopathological factors.

**Variables**	**Total**	**Immune scores**		**P**	**Stromal scores**		**P**
		**High**	**Low**		**High**	**Low**	
Gender							
Male	139	66	73	0.469	75	64	0.285
Female	370	189	181		180	190	
Age (years)							
<45	233	116	117	0.897	113	120	0.507
≥45	276	139	137		142	134	
Histological Type							
Classical	365	197	168	0.000	191	174	0.001
Follicular	102	29	73		36	66	
Tall cell	35	26	9		25	10	
Other	7	3	4		3	4	
Clinical stage							
I+II	339	158	181	0.018	161	178	0.095
III+IV	168	97	71		93	75	
T stage							
T1+T2	310	144	166	0.039	148	162	0.183
T3+T4	197	110	87		106	91	
Lymph node metastasis							
Yes	233	136	97	0.029	130	103	0.070
No	226	109	117		107	119	
Distant metastasis status							
Yes	9	4	5	0.554	3	6	0.174
No	283	154	129		159	124	

**Figure 1:**
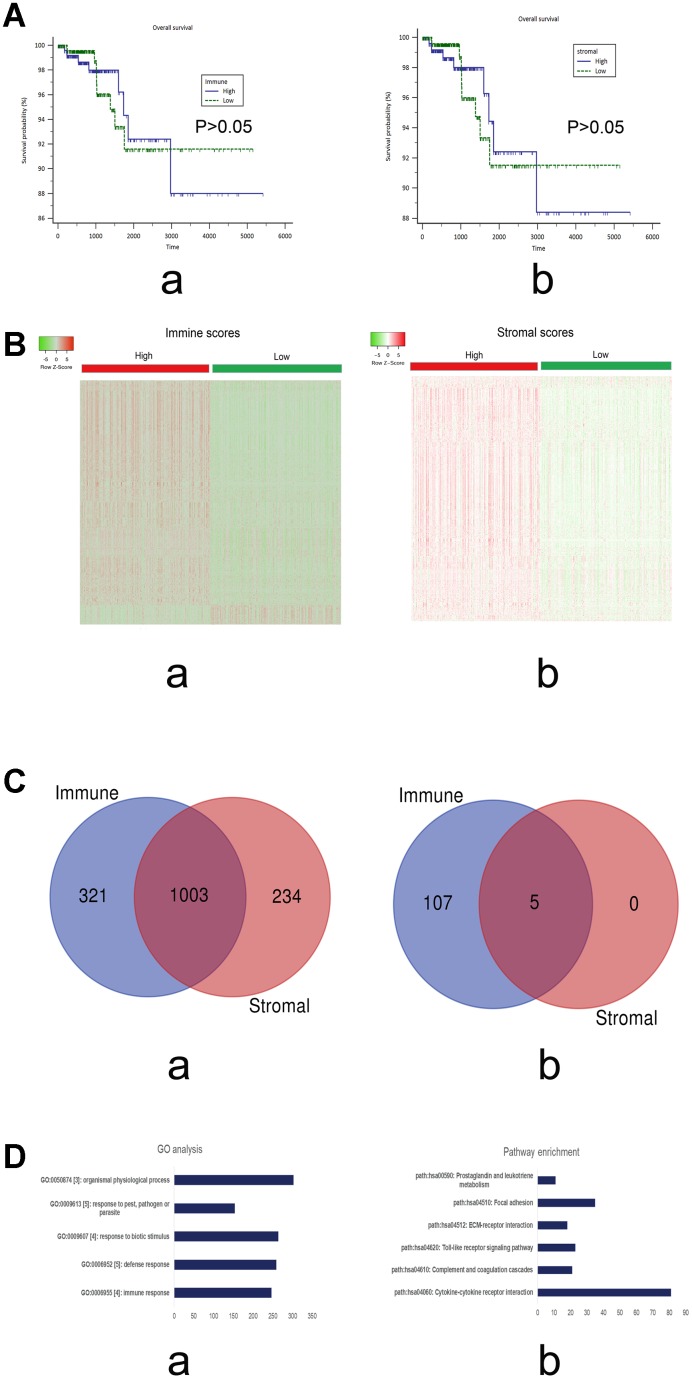
(**A**) Survival curves of the immune and stromal scores based on the TCGA cohorts of PTC. a) immune scores (P>0.05); b) stromal scores (P>0.05). (**B**) Heatmap of the DEGs of immune scores (a; high vs low) and stromal scores (b, high vs low). P<0.05; Fold change>2. Red stands for up-regulated genes, while green stands for down-regulated genes. (**C**) The intersections of the up-regulated (a) and down-regulated genes (b), respectively, from the immune and stromal gene sets. (**D**) The top 5 GO terms (a) and KEGG pathways (b) of the DEGs in the intersections.

Survival curves based on the TCGA cohort were constructed to evaluate the prognostic values of the immune and stromal scores. The results of log-rank tests failed to show that either immune or stromal scores influenced the prognosis of PTC (P>0.05; Figure 1A).

### Identification of differentially expressed genes (DEGs) related to immune scores and stromal scores in PTC

Immune and stromal scores were used to stratify patients with PTC into high and low groups, respectively, as mentioned above. The gene expression profiles were compared between the high and low groups. Heatmaps in Figure 1B show the results generated from the comparisons. The comparison based on immune scores (high vs. low) revealed 1324 upregulated and 112 downregulated genes. Similarly, the comparison based on stromal scores (high vs. low) generated 1237 upregulated and 5 downregulated genes.

Moreover, Venn analysis was performed to narrow the scope of the target genes that are associated with immune and stromal cells. The data showed 1003 upregulated and 5 downregulated DEGs in the intersection (Figure 1C). Thus, these genes were chosen for further analysis.

### Functional annotation of DEGs in the intersection

Gene Ontology (GO) and Kyoto Encyclopedia of Genes and Genomes (KEGG) pathway enrichment analyses were utilized to evaluate the possible functions of the screened DEGs in the intersection.

As shown in Figure 1D, the top five GO terms were immune response, defense response, response to biotic stimulus, response to pest, pathogen or parasite, and organismal physiological process. The top five pathways were cytokine–cytokine receptor interaction, complement and coagulation cascades, Toll-like receptor signaling pathway, ECM–receptor interaction, and focal adhesion.

The results of GO analysis showed that these DEGs might be involved in various cell biological processes, including responses to stimulus and immune response. The data were consistent with our results that the screened DEGs may be correlated with immune responses. Pathway enrichment analysis showed that these DEGs may be enriched in pathways related to cancer development, suggesting that these genes function in immune response and cancer progression.

### Prognostic values of individual DEGs in PTC

Univariate cox regression analyses of the TCGA cohort were conducted to explore the prognostic values of the screened DEGs in the intersection. Of the 1008 DEGs that were assessed, 87 predicted favorable or unfavorable prognosis for PTC (P<0.05; Table 3).

**Table 3:** Univariate Cox regression analyses of the DEGs in the intersection (top 20, ordered by ascending P values).

**Gene symbols**	**HR[exp(coef)]**	**coef**	**95% CI lower**	**95% CI upper**	**Z**	**P**
**NPTX1**	1.451225	0.372408	0.192815	0.552001	4.064233	4.82E-05
**IBSP**	1.493858	0.401362	0.184397	0.618328	3.625713	2.88E-04
**PCOLCE2**	1.534138	0.427969	0.172976	0.682961	3.289516	0.001004
**HAS1**	1.343366	0.295178	0.118928	0.471429	3.282477	0.001029
**GPR34**	0.502837	-0.68749	-1.09996	-0.27502	-3.26681	0.001088
**MEG3**	1.361818	0.308821	0.115307	0.502334	3.127831	0.001761
**BHLHE22**	1.389252	0.328765	0.097692	0.559839	2.788584	0.005294
**FOLR2**	0.526309	-0.64187	-1.10451	-0.17922	-2.71922	0.006544
**PRR15**	0.809274	-0.21162	-0.36701	-0.05623	-2.66914	0.007605
**ETV7**	0.678965	-0.38719	-0.67572	-0.09865	-2.63005	0.008537
**LRRN4CL**	1.426836	0.35546	0.088732	0.622187	2.611987	0.009002
**ICAM4**	0.741666	-0.29886	-0.52444	-0.07328	-2.59662	0.009414
**P2RY13**	0.582569	-0.54031	-0.94957	-0.13104	-2.58752	0.009667
**HS3ST3A1**	1.494091	0.401518	0.096599	0.706437	2.580882	0.009855
**GPR114**	0.693675	-0.36575	-0.64453	-0.08697	-2.57143	0.010128
**DRP2**	1.47486	0.388563	0.088036	0.68909	2.534112	0.011273
**TLR7**	0.613546	-0.4885	-0.86668	-0.11032	-2.53174	0.01135
**LIPH**	0.833861	-0.18169	-0.32284	-0.04054	-2.52287	0.01164
**RXRG**	0.838276	-0.17641	-0.31359	-0.03922	-2.52035	0.011724
**LOC400696**	0.565515	-0.57002	-1.01469	-0.12534	-2.51243	0.01199

### Construction of co-expression network and identification of hub genes associated with LNM

A total of 1008 DEGs in the intersection were included in the co-expression network analysis. All samples were selected because no outliers were observed among the samples.

**Figure 2:**
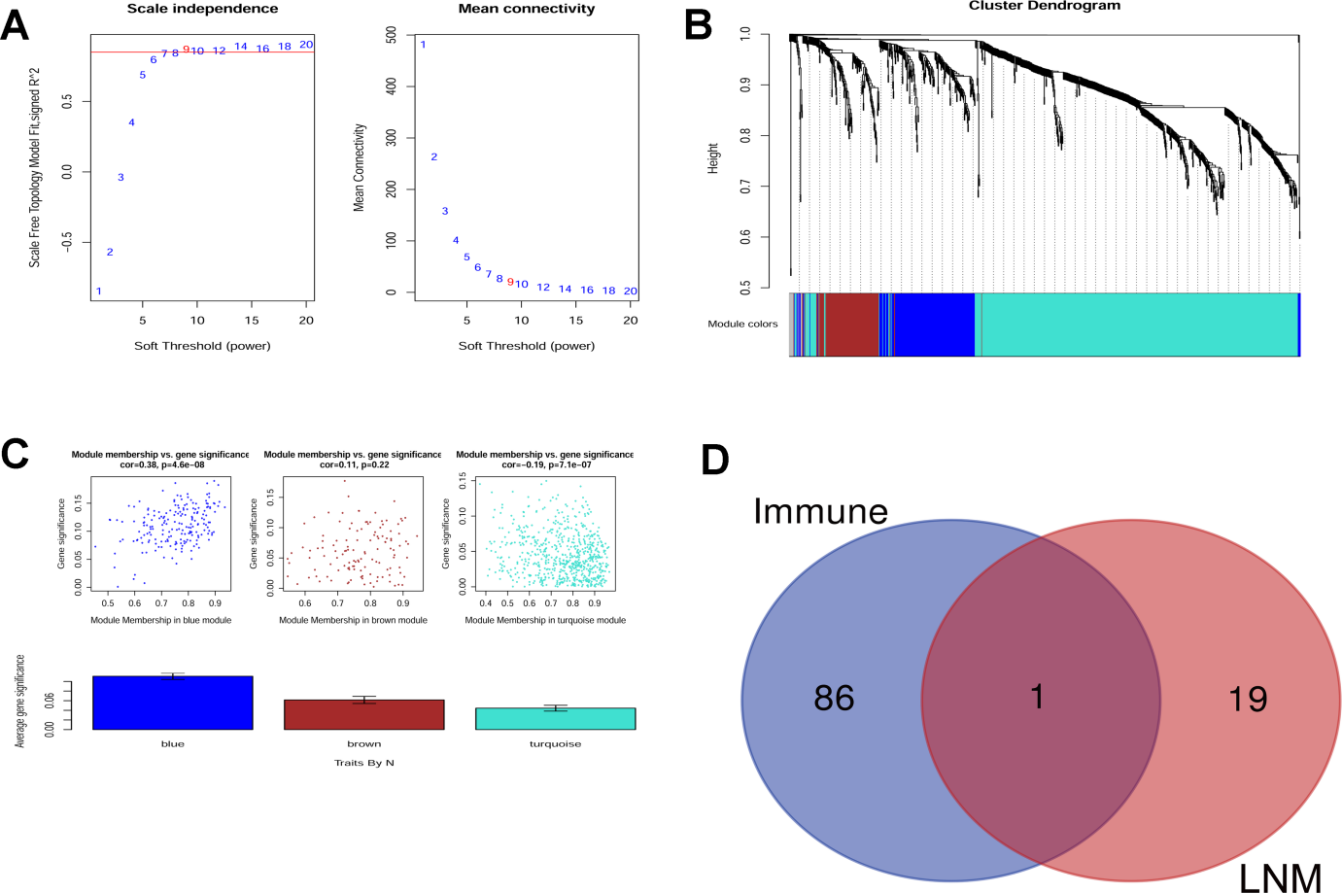
(**A**) Scale independence and mean connectivity analysis. (**B**) Gene clustering tree (dendrogram) obtained by hierarchical clustering of adjacency-based dissimilarity. (**C**) Module-trait relationship plot shows that the blue module has a close correlation with LNM. (D) Venn analysis generated CLDN10 as the key gene that correlates both with LNM and immune.

The soft threshold was determined by scale independence and mean connectivity analysis of modules with different power values. As shown in Figure 2A, when the power value was set to 9, the scale independence value achieved 0.9 and lower mean connectivity. Figure 2B shows the cluster dendrogram among the modules. Four modules containing 194 genes in blue, 126 in brown, 15 in grey, and 671 in turquoise were generated with different colors.

The module that had a relationship with LNM was evaluated. As shown in Figure 2C, the blue module may have the closest association (r=0.38, P=4.6e-08). Thus, the genes in the blue module were selected for further analysis.

### Key gene selection

The following steps were carried out to screen the immune-related and LNM-related key genes in PTC. First, 87 genes that had prognostic values for PTC, as shown in Table 3, were selected (immune-related). Second, the genes in the blue module (LNM-related), with a GS value more than 0.15, were selected (20 genes). Third, Venn analysis was conducted to obtain the intersection of these genes. As a result, CLDN10 was screened as the key gene. Hence, the key gene might be immune- and LNM-related (Figure 2D).

### Assessment of CLDN10 expression in a PTC cohort from the TCGA and gene expression omnibus (GEO) databases

The expression data of CLDN10 in a PTC cohort were extracted from the TCGA database. The expression levels of CLDN10 were classified as high and low on the basis of the median level. Table 4 lists the main results of the association of CLDN10 expression and the clinical features.

**Table 4:** Relationship between CLDN10 expression and clinicopathological factors.

**Variables**	**Total**	**CLDN10**		**P**
		**High**	**Low**	
Gender				
Male	139	70	69	0.942
Female	370	185	185	
Age (years)				
<45	233	129	104	0.029
≥45	276	126	150	
Histological Type				
Classical	365	212	153	0.000
Follicular	102	9	93	
Tall cell	35	29	6	
Other	7	5	2	
Clinical stage				
I+II	339	163	176	0.197
III+IV	168	91	77	
T stage				
T1+T2	310	142	168	0.021
T3+T4	197	111	86	
Lymph node metastasis				
Yes	233	158	75	0.000
No	226	81	145	
Distant metastasis status				
Yes	9	4	5	0.554
No	283	154	129	

As shown in this table, high expression may have a significant correlation with low ages, high T stages, and LNM (P<0.05).

**Figure 3:**
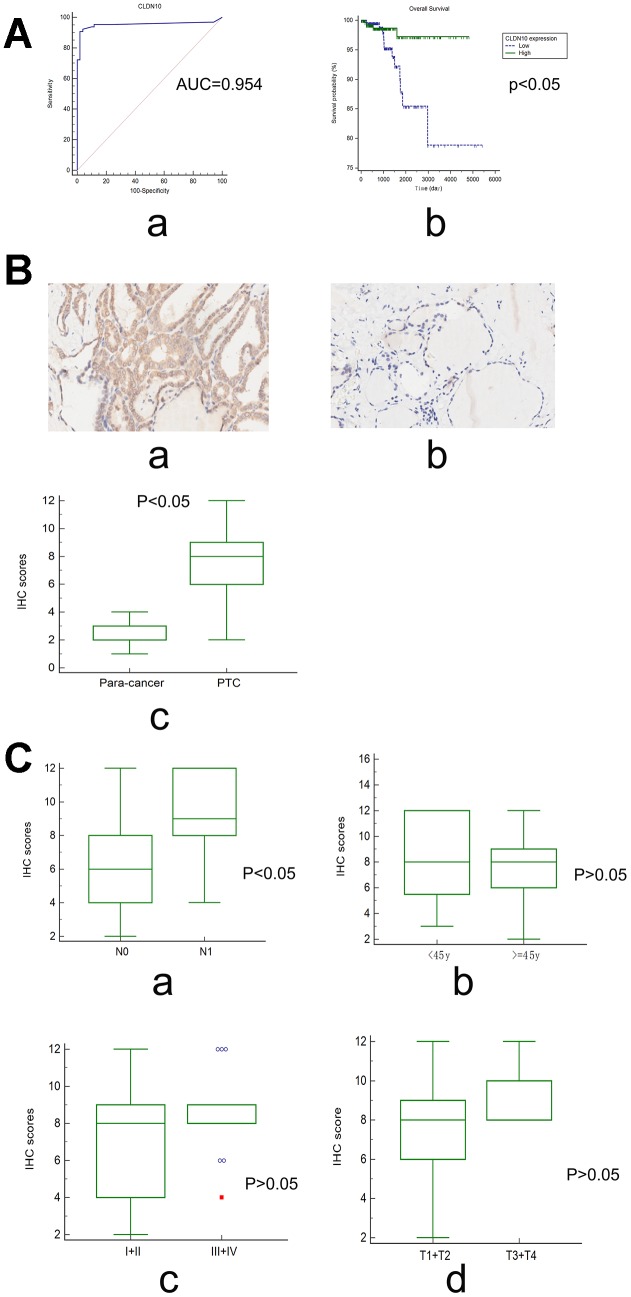
(**A**) The ROC curve (a) showed that CLDN10 expression can be used as a biomarker distinguishing PTC from normal thyroid tissues (GSE35570; AUC=0.954 (95%CI=0.899-0.984); Specificity=0.9804, Sensitivity=0.9077). The survival curve (b) showed that PTC patients with high CLDN10 expression levels had a longer overall survival time relative to those with low CLDN10 expression ones (P<0.05). (**B**) CLDN10 expression in PTC tissues and adjacent normal tissues assayed by IHC (×10). (a) cancer tissue; (b) para-cancer tissue; (c) cancer vs normal, P<0.05. (**C**) The association of CLDN10 protein expression levels with clinical features based on a tissue chip. (a) LNM, N0 vs N1, P<0.05; (b) Age, <45y vs >=45y, P>0.05; (c) Clinical stage, I+II vs III+IV, P>0.05; (d) T stage, T1+T2 vs T3+T4, P>0.05.

Relevant data from a cohort in a dataset (GSE35570) were downloaded from the gene expression omnibus (GEO) database to evaluate the diagnostic value of CLDN10 expression in PTC [12]. This cohort included 65 PTC cases and 51 normal controls. As shown in Figure 3A-a, the area under the ROC curve (AUC) of the receiver-operating characteristic (ROC) curves achieved 0.954, with a sensitivity of 0.9077 and a specificity of 0.9804, suggesting that CLDN10 can be used as a potential biomarker distinguishing PTC cases from normal controls.

Surprisingly, survival analysis showed that patients with low CLDN10 expression may have a shorter overall survival time than those with high CLDN10 expression (Figure 3A-b). In other words, CLDN10 might act as a preventive factor for PTC.

### Protein expression of CLDN10 in a PTC cohort on the basis of a tissue microarray

The association of CLDN10 with LNM was further validated by measuring its protein expression on a tissue chip through an immunohistochemical (IHC) assay.

Specific staining was observed mainly in the membrane of cancer and normal cells, and weak staining was observed in cytoplasm (Figure 3B-ab). In addition, the expression scores of CLDN10 were significantly higher in PTC tissues than in para-carcinoma tissues (P<0.05), as shown in Figure 3B-c.

In this cohort, no cases presented distant metastasis, and the survival information was unavailable. Only the confounding factors, such as clinical stages, lymph node metastasis, and age, can be addressed.

Interestingly, the data indicated that the high expression score of CLDN10 was significantly associated with LNM (P<0.05). No significant associations were observed in the comparisons regarding age, T stage, and clinical stage (P>0.05) (Figure 3C).

### Association of CLDN10 expression with immune cell infiltration levels

The TIMER algorithm [13] was used to determine the possible association between CLDN10 expression and immune cell infiltration.

**Figure 4:**
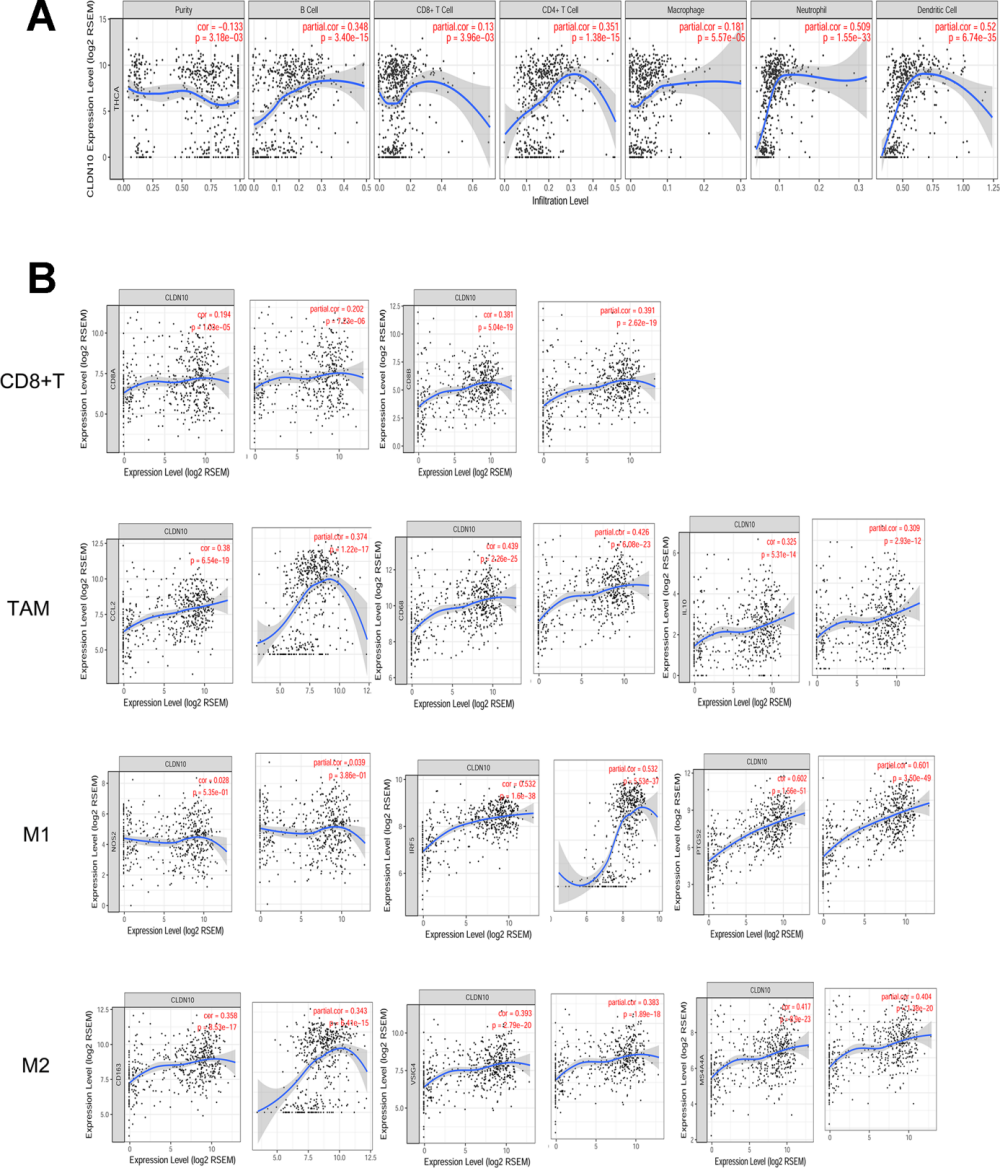
(**A**) Correlation of CLDN10 expression with tumor purity and immune cell infiltration levels in thyroid cancer. CLDN10 expression was negatively correlated with tumor purity (r=-0.133, P<0.05), but positively correlated with B cell (r=0.348, P<0.05), CD8+T cell (r=0.13, P<0.05), CD4+T cell (r=0.351, P<0.05), Macrophage (r=0.181, P<0.05), Neutrophil (r=0.509, P<0.05), and Dendritic cell (r=0.52, P<0.05), respectively. (**B**) The CLDN10 expression was positively correlated with cell markers of CD8+T cell, Tumor-associated macrophage (TAM), M1 macrophage, and M2 macrophage, respectively.

On the basis of the PTC cohort from TCGA, a weak negative correlation was observed between CLDN10 expression and tumor purity (r=-0.133, P<0.05). Conversely, CLDN10 expression had significantly positive correlations with infiltrating levels of B cells (r=0.348, P<0.05), CD4+T cells (r=0.351, P<0.05), neutrophils (r=0.509, P<0.05), and dendritic cells (r=0.52, P<0.05) (Figure 4A). Moreover, weak positive correlations of CLDN10 expression with CD8+T cells (r=0.105, P<0.05) and macrophages (r=0.181, P<0.05), respectively, were observed. These findings indicate that CLDN10 is closely associated with immune cell infiltration in PTC.

Multivariate COX regression analyses were performed using the TIMER tool to learn the potential effects of these immune cells on PTC survival. As shown in Table 5, tumor purity was correlated with poor survival, confirming that CLDN10 expression was negatively correlated with tumor purity. Notably, CD8+T cells (HR=0.000, 95%CI= 0.000–0.032) and macrophages (HR=0.000, 95%CI= 0.000–0.152) might be independent favorable prognostic indicators that lower the cancer risk and prolong the overall survival time of patients.

**Table 5:** Multivariate analyses of CLDN10 expressions and other clinical prognostic markers as well as immune cells related to overall survival in PTC.

**PTC**	**Coef**	**HR**	**95%CI_l**	**95%CI_u**	**P value**
Tumor purity	4.384	80.154	3.887	1.652799e+03	0.005
age	0.174	1.190	1.106	1.282000e+00	**0.000**
gendermale	0.071	1.074	0.267	4.313000e+00	0.920
raceBlack	15.741	6856360.714	0.000	-	0.999
raceWhite	15.535	5579115.168	0.000	-	0.999
B-cell	0.205	1.228	0.000	6.304123e+04	0.970
CD8+Tcell	-18.440	0.000	0.000	3.200000e-02	**0.016**
CD4+Tcell	8.645	5681.442	0.254	1.270458e+08	0.091
Macrophage	-31.546	0.000	0.000	1.520000e-01	**0.037**
Neutrophil	-33.653	0.000	0.000	5.740182e+10	0.259
Dendritic	11.912	149010.289	0.557	3.989821e+10	0.062
CLDN10	-0.138	0.871	0.727	1.044000e+00	0.135

### Association of CLDN10 expression with immune cell markers

Markers of immune cells were considered to investigate further the association of CLDN10 expression with CD8+T cells and macrophages. As shown in Figure 4B and Table 6, CLDN10 expression was correlated with immune markers of macrophages and CD8+T cells. In consideration that macrophages and CD8+T cells might be favorable prognostic indicators for PTC, the data may help explain why low CLDN10 expression is related to poor prognosis in patients with PTC.

**Table 6:** Correlation between CLDN10 and related gene markers of relevant immune cells.

**Immune cell**	**Gene Markers**	**None**			**Purity**	
		**r**	**P**		**r**	**P**
CD8+T cell	CD8A	0.194	0.000		0.202	0.000
	CD8B	0.381	0.000		0.391	0.000
TAM	CCL2	0.380	0.000		0.374	0.000
	CD68	0.439	0.000		0.426	0.000
	IL10	0.325	0.000		0.309	0.000
M1 Macrophage	INOS(NOS2)	0.028	0.535		0.039	0.386
	IRF5	0.532	0.000		0.532	0.000
	COX2(PTGS2)	0.602	0.000		0.601	0.000
M2 Macrophage	CD163	0.358	0.000		0.343	0.000
	VSIG4	0.393	0.000		0.383	0.000
	MS4A4A	0.417	0.000		0.404	0.000

## DISCUSSION

A discovery phase and two validation phases were included in the present study. In the discovery phase, CLDN10 was chosen as the key immune-related and LNM-related gene. In the validation phases, high CLDN10 expression was shown to correlate with LNM in PTC at the mRNA and protein levels. It had a positive correlation with infiltration of various immune cells. Surprisingly, high CLDN10 expression may predict a good prognosis rather than a poor prognosis in patients with PTC. The discrepancy of the results may distort our conventional view that LNM-related genes are correlated with poor prognosis in cancers.

CLDN10 encodes Claudin-10, an integral tight junction membrane-spanning protein expressed in the kidney, skin, and salivary glands [14]. Abnormal expression of CLDN10 may be involved in cancer progression. For instance, overexpression of CLDN10 in osteosarcoma may be related to its metastatic phenotype [15]. In liver cancer, CLDN10 overexpression may be correlated with cell proliferation and invasive abilities [16]. Nevertheless, no difference in CLDN10 expression was observed between laryngeal cancer tissues and normal controls [17]. Therefore, CLDN10 might serve different functions in different malignant tumors.

A recent report [18] has shown that high CLDN10 expression is correlated with LNM in PTC patients on the basis of the TCGA cohort, which is in line with the present study. However, this report has also presented that patients with high CLDN10 expression experience a poor overall survival, which is contradictory to our results. Thus, we have endeavored to repeat the survival analysis on the basis of the data from TCGA and a used the UALCAN and TIMER tools. However, the results still indicated CLDN10 as a favorable prognostic indicator rather than an unfavorable indicator for PTC.

The discrepancy was confusing to an extent. The associations of CLDN10 with immune cell infiltration in PTC were further assessed to explain the objective phenomenon of this contradiction. CLDN10 showed a positive correlation with the immune cells, such as CD4+T cells and macrophages, and a negative correlation with tumor purity. Increased CD8+T cells and macrophage infiltration predicted better prognosis, as indicated by the Cox regression analysis. Thus, increased immune cell infiltration might play a leading role in the survival of patients, although LNM might also have a certain impact on survival.

Previous evidence showed that the presence of LNM in PTC predicts poor prognosis [19]. Conversely, recent evidence has shown that LNM is not a good predictor for PTC prognosis because the microenvironment of LNM presents features that favor an anti-tumor immune response [20]. In specific, infiltration of CD8+T cells may protect against metastatic spread in PTC [21], and a high infiltration of CD68+ cells (macrophages) in tumor stroma may predict long survival time [22]. In addition, the animal experiments confirmed the anti-tumor activities of CD8+T cells and M1 macrophages in PTC [23]. Thus, the weight of immune cell infiltration on prognosis may be greater than that of LNM in PTC. The evidence may help clarify the reasons why high CLDN10 expression acts as a favorable prognostic factor for PTC, although it has a significant correlation with LNM.

In the present study, the ESTIMATE Algorithm was used to screen immune-related genes, and the WGCNA method was used to screen LNM-related genes in PTC. The intersection involved CLDN10 as the key gene. Aside from ESTIMATE and TIMER, other algorithms (such as CIBERSORT) can be used for immune cell infiltration [24]. However, each algorithm has its own advantages and disadvantages, which have been verified by experiments. None of the algorithms is the most authoritative. Therefore, we adopted the ESTIMATE and TIMER algorithms in the present study to understand the infiltration of immune cells. WGCNA was applied to investigate the relationship between co-expression gene modules and clinical traits. In the present study, we successfully identified CLDN10 as the immune-related and LNM-related gene in PTC, demonstrating WGCNA as a good method for gene screening.

Although the possible functions of CLDN10 have been indicated in our study, future lab experiments using cell lines and animal models are still needed to explore its exact mechanisms. Future studies using tissue chips containing a large PTC cohort with survival information are also warranted to confirm the effects of CLDN10 expression on PTC prognosis.

In conclusion, CLDN10 was screened as a key immune-related and LMN-related gene in PTC. Although the high expression of CLDN10 is associated with lymph node metastasis, it provided better predictions for PTC. The discrepancy might be due to the preponderant weight of the CLDN10-related infiltration of CD8+T cells and macrophages on the prognosis, suggesting immune cell infiltration as a negligible factor in the development of PTC. The data of the present study suggest that LNM-related proteins might not necessarily predict unfavorable outcomes in cancers. The effect of tumor purity and immune cell infiltration on prognosis should also be considered in cancer research.

## MATERIALS AND METHODS

### Data source

A cohort of PTC was retrieved from the TCGA database [25]. The gene expression profile based on RNA-seq and relevant clinical data were downloaded from the TCGA and UCSC Xena databases, respectively [26].

The stromal and immune scores in the PTC TCGA dataset were calculated based on the ESTIMATE algorithm to predict the level of infiltrating stromal and immune cells in PTC and infer tumor purity in tumor tissue [27]**.**

The data were analyzed by using the package limma of R program (version 3.6.0). The samples were divided into two groups (high and low) on the basis of the immune and stromal scores, and the two groups were compared. The results were downloaded and analyzed, in which the genes that met the cut-off criteria of adjusted P<0.05 and a |log fold-change| of >1.0 were screened.

The screened genes regarding immune and stromal scores were processed with Venn analysis. Genes of the intersection were considered as the DEGs.

### Functional annotation of the DEGs

GO and KEGG pathway enrichment analyses were conducted by using the Gather database to learn the possible functions of the DEGs [28].

GO is a comprehensive community-based bioinformatics resource that provides information about gene or gene product function using ontologies to represent biological knowledge [29]. In the GO analysis, the gene network was presented according to biological processes [30]. KEGG is a reference knowledge base for linking sequences to biological functions from molecular to higher levels [31], which is an integrated database consisting of three generic categories of systems information, genomic information, and chemical information [32].

A *P*-value less than 0.05 was considered statistically significant.

### Construction of weighted co-expression network (WGCNA) and identification of modules related to LNM

Relevant gene modules were identified using the WGCNA method to screen the possible gene sets associated with LNM [33]. WGCNA is an algorithm widely used in gene co-expression network identification in a number of disorders to find significantly correlated gene modules. Scale independence and mean connectivity analysis of modules with different power values were conducted to assess the soft threshold of module analysis. The power value was determined when the scale independence value was 0.9. Then, with the minimal module size of 30 and the merge cut height of 0.25, the co-expression matrix was calculated. The results of dynamic tree cut and merge were displayed by clustering dendrogram.

Information regarding the clinical features was used to identify significant co-expression modules associated with the clinical traits. The related gene modules concerning clinical features (such as LMN) were identified by calculating their correlations. Module membership (MM) and gene significance (GS) were calculated in an intramodular analysis of module statistically. Significant modules were defined with a P value less than 0.05.

### Key gene screening

The expression levels of the DEGs were divided into high and low groups on the basis of their median levels. From the TCGA data, the univariate cox regression analyses were used to evaluate their prognostic values for PTC. The genes with P values less than 0.05 were selected. Second, the modules that had a close association with LNM were chosen. The genes with a GS of more than 0.15 were selected. Third, Venn analysis was conducted to filter the intersection of the genes selected from the above two steps.

### PTC specimens

A PTC tissue microarray (HThy-Pap120CS-01) comprising 58 PTC tissues and 58 paired adjacent non-cancer tissues was purchased from Shanghai Outdo Biotech Co., Ltd.. All patients were pathologically diagnosed as PTC and received no extra treatment before surgery.

### IHC staining and evaluation

The protein expression levels of the key genes were tested by using the two-step method of IHC as previously described in our study [34]. In brief, the slides were deparaffinized, rehydrated, and treated with 3% hydrogen peroxide for 20 min to inhibit endogenous peroxidase. The sections were rinsed with distilled water and saturated in phosphate buffered saline for 5 min and then incubated with a 1:200 dilution of rabbit anti-polyclonal antibody (primary antibody; Abcam) overnight at 4 °C. The staining was visualized using DAB solution and counterstained with hematoxylin. IHC staining was conducted in accordance with the manufacturer’s instructions.

The IHC stain results were identified by integrated scoring. The results were evaluated and scored independently by two pathologists without knowledge of the clinical parameters of the cases. The staining intensities of the proteins were scored from 0 to 3, where 0 means negative, 1 weak, 2 moderate, and 3 strong. The percentages of positively stained cells were scored in scales of 0 to 4, in which 1 represents (0–25%), 2 (26%–50%), 3 (51%–75%), and 4 (76%–100%). The proportion and intensity scores were then multiplied to gain a total score, with a range from 0 to 12.

### Statistical analysis

For continuous variables, differences between the groups were analyzed using ANOVA, a *t*-test, or a Wilcoxon rank sum test in accordance with the concrete types of the data. Chi-squared test was used to differentiate the rates of different groups. The diagnostic accuracy of the genes was measured by the ROC curves and the AUC. The Kaplan–Meier method was conducted to calculate the overall survival curves. A log-rank test was used to determine differences in the survival rates. These analyses were performed by utilizing MedCalc software (15.2.2; Mariakerke, Belgium). Statistical significance was considered at *P* < 0.05.

### Ethical conduct of research

The authors state that they have obtained appropriate institutional review board approval or have followed the principles outlined in the Declaration of Helsinki for all human or animal experimental investigations. The present study used a commercial tissue microarray that was purchased from Shanghai Outdo Biotech Co., Ltd.
